# A Study on the Preference for the Defensive Attribute under Environmental Risk in China

**DOI:** 10.3390/healthcare8010047

**Published:** 2020-02-27

**Authors:** Xiaohong Chen, Li Zhou

**Affiliations:** College of Economics and Management, Nanjing Agricultural University, Nanjing 210095, China; 2017206014@njau.edu.cn

**Keywords:** defensive health behavior, environmental pollution, risk perception, drinking water safety, discrete choice experiment, social networks

## Abstract

Little is known about the preference for defensive consumption goods and their defensive attributes under environmental risks in developing countries. The paper takes the water purifier as an example of defensive consumption goods against heavy metal pollution risk from drinking water. Using a survey data in China, the discrete choice experiment method is employed to investigate rural farmers’ preferences for a water purifier. The scientific knowledge and risk perception effects are used to determine farmers’ preferences for the defensive attribute on a water purifier. Using a mixed logit model, rural farmers are found to prefer a water purifier with low price, free installation, a longer warranty period, and a heavy metal filter (i.e., the defensive attribute). Farmers’ neighborhood norm perception dominates the defensive preference while scientific knowledge do not work significantly. More specifically, the more the neighborhood norm perception is recognized, the more likely farmers are to improve their own preferences for the defensive attribute. Affected by the neighborhood norm perception, rural farmers’ preferences for the defensive attribute are found to be increased significantly as average income gaps narrow. The future defensive health policies should be devoted to improving environmental risk awareness and utilizing informal social networks in areas with high environmental risks.

## 1. Introduction

Previous studies have rarely considered the preference for defensive consumption goods and those defensive attributes under environmental risks. Those goods purchased in defensive behaviors to reduce pollution exposure and prevent against environmental damage can be regarded as the defensive consumption goods. Constructing effective public policies to deal with environmental and health hazards requires understanding how an individual’s preference for defensive consumption goods responds to changes in risk. This paper intends to discuss this issue using a discrete choice experiment (DCE) method toward frequent heavy metal pollution warnings in developing countries.

Although China, the largest developing country, has witnessed one after another heavy metal pollution events during rapid industrialization, very little has been done to directly examine residents’ preferences for defensive consumption goods under heavy metal pollution risk. This information is critical for policy makers to perform analysis of socially efficient externality and potential risk mitigation strategies. Previous studies have concentrated more on demographics, knowledge, and risk perception to illustrate people’s environmental risk awareness and responsive behaviors. The models including knowledge, risk attitude, and perception are more general in nature [1], and there is still a lack of an analysis framework to link them with preference for defensive consumption goods. Therefore, this study aims to explore the role of scientific knowledge and risk perception played in preference decisions on the defensive attribute under heavy metal pollution risk.

As Chinese consumers are increasing their demand for food safety attributes [2], some defensive attributes for drinking water safety may be of concern gradually. The common avoidance behaviors of drinking water include the use of water treatment equipment, such as the water purifier rather than tap water [3,4,5]. Thus, the water purifier is chosen to examine an individual’s preference especially for its defensive attribute against heavy metal pollution. The paper not only provides guidance for research on the determinants of preference for the defensive consumption goods, but also provides reference for policy makers on social health and welfare improvement, such as the 3rd and 6th sustainable development goals (SDGs) for “good health and wellbeing” and “clean water and sanitation”.

Our research contributes to the existing literature of environmental hazards and defensive consumption behaviors in the following ways. First, while much attention has focused more on defensive actions, little research has been dedicated to analyzing defensive consumption goods and their defensive attributes. For air pollution, people could adjust daily activities to defend against both static and dynamic smog alerts [6,7]. In terms of defensive consumption behaviors, contaminated drinking water or tainted food may also cause adjustments as a result of announced risks. Those households with unsafe water sources are urged to switch to a safer one [8] or make choices between bottled, filtered tap, and unfiltered tap water [3] in response to water contamination risks. Negative information, such as the methyl-mercury hazard, has reduced store-bought fish consumption as defensive behaviors for residents in risky areas [9]. Similar, positive information of food safety policies can promote offsetting behaviors, causing customers to become less alert and eat more unsafe foods [10]. In addition to adjusting consumption behaviors, extra expenditures on defensive consumption goods have been noted by academia. For example, people may take respiratory medications [5] or purchase anti-haze masks [11] when they are exposed to air pollution. Some households may buy air conditioners affected by temperature fluctuations [12]. Unfortunately, the literature on the preference for defensive attribute is limited, especially for the heavy metal pollution hazard. A DCE approach is adopted to fill the gap. A DCE method has been widely used in marketing, ecological economics and health economics [13,14]. The attributes of low price, free installation, longer warranty period, and heavy metal filter are found to be significant factors of preference for a water purifier. The heavy metal filter as the defensive attribute is complementary to the brand and price, but substitutable to free installation.

Second, this study intends to investigate preferences for the defensive attribute under the impact of scientific knowledge and risk perception effects. In addition, the income gap and education gap are speculated as potential moderators of these relationships. The effects of scientific knowledge and risk perception on behavioral decision making are emphasized by previous literature [15,16,17]. An individual’s chance of making mistakes can be influenced by incomplete knowledge when solving problems, because they may choose incorrect strategies [18]. Food labeling knowledge is the most common content. Some studies indicate that environment-related food labels lead consumers to purchase more environmental-friendly food [19,20]. In some cases, the evidence with respect to knowledge and consequent behaviors is ambiguous and mixed. For example, improved knowledge does not have positive effects on the misuse of fertilizers [21] and willingness to buy lottery tickets [16]. Therefore, the effect of scientific knowledge on the preference for defensive consumption goods may remain uncertain. Besides, while public perception plays a critical role in residents’ consumption patterns [4,22,23], the analysis that linked public perception and preference for the defensive attribute has received little attention. Overall, the existing literature does not present a uniform framework to understanding an individual’s decision-making process of the defensive preference. In this paper, some significant positive coefficients for neighborhood norm perception, but not for scientific knowledge, are observed of preference for the defensive attribute. More specifically, as the neighborhood norm perception is more recognized by residents, their preferences for the defensive attribute are more likely to be improved. The preference for the defensive attribute affected by neighborhood norm perception is shown to be significantly enhanced as the average income gap narrows.

The rest of the paper proceeds as follows. The second section introduces the background between heavy metal pollution, water and health, and the choice experiment method, including descriptions of design and data. The third section presents the estimation results of various specifications and robustness checks. Our conclusions and relevant policy recommendations are given at the end.

## 2. Background and Methodology

### 2.1. Background: Heavy Metal Pollution, Water, and Health

**Figure 1:**
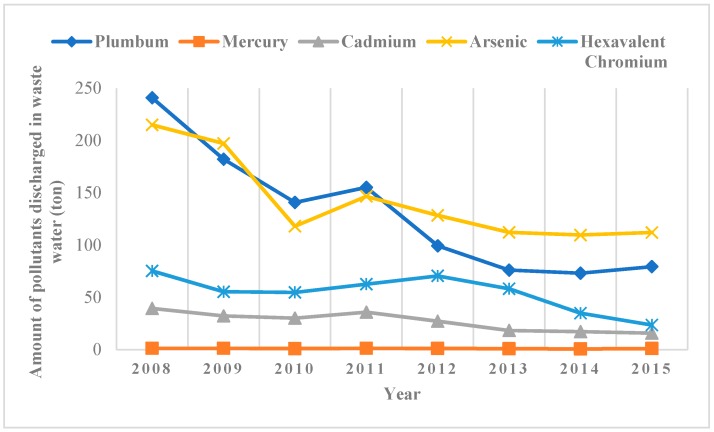
Trends of five heavy metal pollutants emissions in China.

As the Chinese industrial process has rapidly developed, it has become evident that heavy metal pollution emissions are responsible for a series of potential health crises. The discharges of industrial wastewater and waste solid cause severe heavy metal pollution on drinking water sources. As early as 1956, the Japanese Minamata disease event was caused by the discharge of heavy metal polluted wastewater, which was one of eight major public hazards in the world. In China, the Guangxi cadmium river event in 2012 and Hunan cadmium rice event in 2013 had also raised concerns about heavy metal pollution (see reference [24,25]). Figure 1 shows trends of five heavy metal pollutants emissions in China. Although the overall trend of some pollutants tends to decrease, the national discharge amounts of five common heavy metal pollutants only in waste water were 79.43 ton (Lead), 1.08 ton (Mercury), 15.82 ton (Cadmium), 112.10 ton (Arsenic), and 23.598 ton (Hexavalent Chromium), respectively, in 2015.

The Chinese government has issued many policies and measures to control heavy metal pollution on drinking water source. The government has a responsibility to promise drinking water is of good quality, especially odorless, colorless, free of pathogenic microorganisms or unsafe chemicals [23]. In 2010, the first regional heavy metal pollution treatment program, named Comprehensive Treatment Program for Heavy Metal Pollution in Xiangjiang River Basin, was officially approved by the State Council. The goal was to reduce the number of heavy metal enterprises and their heavy metal emissions by 50% compared with 2008 in the Xiangjiang River Basin, China. The latest regulation is called Opinion on Strengthening Pollution Prevention and Control of Heavy Metal Industry that was issued by the Ministry of Ecological Environment in 2018. The goal is to reduce the emissions of heavy metal pollutants from key industries in China by 10% in 2020 compared to 2013. It also focuses on solving a few prominent heavy metal pollution problems that threaten the health welfare and safety of agricultural products. However, these regulations may not fully protect the public from health risks while the government agencies manage to control environmental pollutants [5].

Moreover, the quality of drinking water and current process of water plants on heavy metal removal cannot be well guaranteed in some rural areas, causing potential health risks. Different from organic pollutants, heavy metal pollutants characterized as non-degradable, toxicity, long-lasting, and bioaccumulation do great harm to human body. Once heavy metal pollution exceeds the drinking water quality standard, drinking water safety, and health outcomes of residents may be affected. Take the heavy metal pollutant cadmium as an example, it is usually discharged into the environment through wastewater, and then enters the food by irrigation [26]. The hygienic standard for drinking water in China stipulates that the cadmium shall not exceed 0.005mg/L (GB 5749-2006, China). Even a low dose of cadmium intake is also harmful to health. Cadmium is nephrotoxic and osteotoxic. It can cause kidney tubular damage, kidney failure, and Itai-Itai disease [27,28]. Itai-Itai disease originated from a public nuisance event in Japan caused by the discharge of cadmium-containing wastewater from 1955 to 1977. The increased risk of cancer in the lung, endometrium, bladder, and breast are also found to be relevant to cadmium [28]. Cadmium intake is significantly associated with increased mortality by 20% and non-cardiovascular mortality by 44% [29,30]. The health risk of these pollution-related diseases also drives up private health spending in affected communities, undermining residents’ welfare [31,32].

Overall, the repair and treatment of heavy metal pollution is difficult to complete in a short period of time. Therefore, an individual may adopt corresponding defensive behaviors or buy defensive consumption goods to avoid potential health risks [3]. It is therefore essential and imminent for policy makers to focus on those defensive behavioral decisions and defensive consumption goods of drinking water under heavy metal pollution risk.

### 2.2. Discrete Choice Experiment (DCE) Model

A DCE method was adopted to investigate preference effects for a water purifier with different attributes. The choice experiment is derived from Lancaster’s theory of consumption characteristic value [33]. Based on random utility theory and customer demand theory, survey respondents were asked to perform sequential choices between different combinations. By providing a choice set of different attributes, the method required these respondents to carefully weigh multiple alternatives and make trade-offs to choose one that could maximize their utility. Then, an econometric method was used to evaluate the preference for various attributes. The DCE also can break through the limitations of some traditional methods and produce evaluation results consistent with welfare economics. Besides, it can eliminate or reduce the embedded bias and strategic deviation of contingent valuation method (CVM).

Based on the utility maximization hypothesis, a survey respondent obtains the utility of the scheme *i* among the *n* schemes of a choice set.

(1) $$U_{i}=V_{i}\left(x_{i},s\right)+\varepsilon_{i}$$

In Equation (1), *U_i_* represents the potential utility of scheme *i*; *V_i_ (x_i_, s)* represents the observable utility part, and the utility function *V_i_* can be estimated according to the attribute *x_i_* and characteristics *s*. $\varepsilon_{i}$ is the random error term which represents the non-observable utility part. The observable utility function *V_i_* is usually denoted as follows:

(2) $$V_{i}=ASC_{i}+\sum \beta_{k}x_{k}$$

where Equation (2) is the basic model. ASC is a specific alternative constant, which represents the benchmark utility of “maintain the status quo” or “do not choose”. ASC contains the average utility brought by all other scheme attributes not included in the model [34]. The independent variables are *k* scheme attribute variables *x_k_*, and ($\beta_{1}$, …, $\beta_{k}$) are estimated coefficients. When there is another option *i’*, the probability of a survey respondent choosing option *i* is

(3) $$P_{i}=P[\left(V_{i}+\varepsilon_{i}\right)>\left(V_{i^{\prime }}+\varepsilon_{i^{\prime }}\right)]$$

In Equation (3), if *i* is randomly distributed, Equation (4) belongs to mixed logit model. This model is also called random parameters logit (RPL) model. The mixed logit model can relax the restrictions on independently identically distribution (IID) and independence of irrelevant alternatives (IIA) [35] and capture the preference heterogeneity [13]. The estimated results of a mixed logit model are more consistent with the actual situation, and therefore better than the multinomial logit model. The equation of marginal substitution rate between the defensive attribute and other water purifier attributes can be expressed as the following:

(4) $$MSR_{A}=-\left(\beta_{A}/\beta_{d}\right)$$

where *MSR_A_* is the marginal substitution rate for *Ath* attribute, *β_A_* is the derivative of the utility against the *Ath* attribute, and *β_d_* is the derivative of utility against the defensive attribute. Besides, the complementary effects, substitution effects, and regional heterogeneity of preference for the defensive attribute are also considered as extended analyses.

## 3. Attributes and Experimental Design

To reflect as much realism as possible, attributes and levels were obtained based on a review of drinking water literature, consultation with experts, and market research in survey areas. This paper focuses on the five main attributes of price, brand, free installation, warranty period, and heavy metal filter on the water purifier.

(1) Price (Price). According to the average price of water purifier sales on the market and income level of residents in the survey area, three levels of 1000 CNY, 2000 CNY, and 3000 CNY were included in the design, resulting in a great variation in price.
(2) Brand (Brand). Brand is usually closely related to product quality. There are rich and mixed brands of water purifiers in the market. To be simplified, we set the brand attribute as two levels: international brand and domestic brand.
(3) Free installation (Installation). The installation and application methods of water purifiers are not uniform. In general, the installation cost of water purifier is not very high, while transportation cost should be considered. The installation fees are expected to be higher costs when the shipping area is far from the stores. Accordingly, a two-level attribute was included for the free installation effects: Yes and No.
(4) Warranty period (Repair). The filter type determines warranty period of water purifier, which is between 1–3 years in general. Therefore, three-levels of warranty period were coded as 1 year, 2 years, and 3 years.
(5) Heavy metal filter (RO). Water purifiers have a variety of filters corresponding to different purification functions and pollutants. General filters, such as PP cotton, is an initial filtration of raw water to remove coarse particles of impurities, sludge, colloids, suspended substances, etc. The pore size of reverse osmosis membrane (RO) is 0.1 nm, which removes heavy metal pollutants and other organic impurities from water effectively. Therefore, we specified the heavy metal filter as RO with two levels of Yes and No. Heavy metal filter is the key defensive attribute of this study.

In our design, the attributes of price and warranty period included three levels, respectively. Brand, free installation, and heavy metal filter contained two levels, respectively (see Table 1 in detail). Based on these attributes and level settings, a total of 72 (3 × 3 × 2 × 2 × 2) combination items of attributes could be obtained. Each respondent needs to compare and make choices on water purifier between these combinations, which is not feasible. Considering the acceptability of respondents and investigation time, we used the partial factor design method to apply an orthogonalization procedure in IBM SPSS 23.0 (SPSS Inc., Chicago, IL, USA) software to eliminate unrealistic and strong alternatives. More specifically, eight first-level choice sets (i.e., eight questionnaires) were employed based on the balance principle of attribute levels. Each respondent randomly chose one choice set at the survey time. Each first-level choice set contained three secondary-level choice sets with two schemes and one option of “do not participate in any scheme” (ASC). Some respondents may choose the ASC since they do not pay attention to the proposed environmental change and prefer the status quo of environmental products [34]. If a survey respondent chooses option A, it indicates that the expected utility of option A is higher than that of both option B and option C.

The design had a good level of D-optimality with a D-efficiency of 93.17%. A total of 40 pairs were constructed to 5 blocks and 8 choice sets. To avoid the effect of question order on decision-makings in the DCE, all investigators were instructed to select a scenario in a randomly order. The survey adopted the household surveys of family members engaged in agricultural production and consumption. We used face-to-face interviews guided by some questionnaires. The investigators first administered a set of socio-economic questions regarding personal information, scientific knowledge, and risk perception. Then they carefully explained each attribute level of a water purifier to ensure respondents accurately understood these requirements. All respondents were asked to conduct DCEs on the premise that they could retell the content of schemes after additional explanation. Table 2 is an example of choice sets. The question paradigm asked by all investigators is as follows.

Assuming you purchase a water purifier from the market, each of the following tables contains two types of water purifiers, A and B, while the other characteristics are consistent. Below are some combinations of different attributes of water purifiers. Please choose the one that is most preferred based on your true wishes. If you are not satisfied with both, you can also choose option C-“purchase other water purifiers C other than the A/B option”. The water purifier C is just a filter head, made by PP cotton. It can only remove large particles such as sediment and iron filings from water. RO reverse osmosis membrane in A or B can effectively remove heavy metal pollutants such as cadmium and other harmful substances from drinking water.

## 4. The Scientific Knowledge and Risk Perception Effect

Moreover, the defensive attribute of a water purifier is our main interest. This paper intends to discuss the internal logic of defensive consumption decisions under heavy metal pollution risk. First, scientific knowledge of heavy metal pollution hazard may determine the preference for the defensive attribute. Enough knowledge enables people to make a comprehensive judgment and reasonable decision on their own environmental risks [1]. Scientific knowledge is found to be a determinant of personal judgment on health food [36], and it can promote the purchase of green products [37] and decrease environmental health burdens [38]. Based on the mixed logit model, acquired scientific knowledge variables are interacted with the heavy metal filter to estimate preference for the defensive attribute respond to scientific knowledge effects. We take cadmium pollutant as a study example. The scientific knowledge effects are measured by three question dummies related to the cadmium risk, including sources of cadmium pollution (source), ways in which the body ingests cadmium (intake), and diseases caused by cadmium (disease).

Second, while scientific knowledge may improve people’s understanding of environmental hazards, risk perception may also be another decisive factor to illustrate their risk prevention awareness. Previous literature concluded that negative risk perception of drinking water sources is a key factor for residents to choose water purifiers [3], such as health concerns [4]. Massoud et al. [23] also noted that the perception of bottled water affects drinking water preferences and consumption patterns since bottled water has a better quality. Even consumers’ risk perceptions and attitudes are responsible for offsetting behaviors associated with positive information provided by food safety policies [10].

We construct risk perception variables with reference to the theory of planned behavior (TPB). Based on rational behavior theory in 1988 and 1991, Ajzen added some pre-factors that can affect an individual’s willingness to act and predict the certain behaviors to the TPB [39]. According to TPB, an individual’s actual behavior is directly guided by the behavior intention. The attitudes, subjective norms, and perceived behavioral controls indirectly affect actual behavior through behavior intentions. TPB has been widely used in various fields of behavioral economics [40]. The risk attitude in TPB refers to an individual’s evaluation and definition of a specific behavior formed by attitude conceptualization. We construct a risk attitude perception by perceived risk using “cadmium in your drinking water exceeds the safety standard”, which is recorded as attitude. The subjective norm in TPB refers to the social (or influential decision makers) pressure one feels when making a behavioral decision. We consider a neighborhood norm perception measured by “you have relatives, friends, and neighbors that think that you should buy a water purifier with the heavy metal filter to reduce health risks of cadmium”, which is recorded as norm. Some people may follow the other’s thoughts or behaviors by the lack of their own opinions, which will directly affect consumption preferences and decision-making results [41]. The perceived behavioral control in TPB refers to the hindrances of an individual’s past experiences and expectations. When people think that the more resources and opportunities they have, and the fewer obstacles they expect, the more behavioral control they perceive. We use “household water consumption decision is up to you” to measure the behavioral control perception, which is recorded as control. The more people agree with the above three viewpoints, the stronger their risk perception is. Further research has demonstrated that risk attitudes, subjective norms, and perceived behavioral controls can impose significant effects on a consumer’s behaviors both directly and indirectly [15,37,42]. Three interaction terms between risk perception variables and the heavy metal filter are employed to capture heterogeneous preferences for the defensive attribute response to risk perception effects.

**Figure 2:**
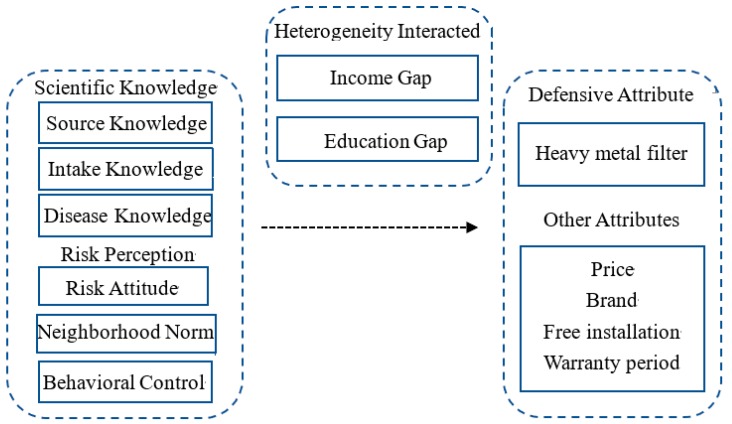
Analytical framework.

The preference for defensive attribute affected by scientific knowledge and risk perception may also interact with average income gaps and education gaps. The inherent bias of information processing and memorizing accounts for non-normative decision-makings [36]. Compared with general attitudes, risk perception also requires a better understanding of the issue [1]. Those people with better educational background and more information sources related to income level have a stronger ability to understand and analyze information [5,43]. Some studies have highlighted that education attainment is significantly associated with residents’ environmental risk awareness [31], which may help to make defensive decisions respond to heavy metal pollution risk. Higher wages are found to increase the likelihood of engaging in defensive behaviors due to their additional costs [5]. Low-income people are less aware of environmental risks [44], and rely more on their immediate social networks of family and friends rather than official information sources in response to health risks [45]. Overall, income gaps (Incgap) and education gaps (Edugap) between each respondent and average village level are utilized to illustrate the scientific knowledge and risk perception effects on the heterogeneous preferences for the defensive attribute. The specific variable definitions are shown in Table 3. Based on the above analysis, Figure 2 presents the analytical framework.

## 5. Data and Summary Statistics

Some studies have pointed out that rural residents are more vulnerable to environmental change due to their resource constraints [12]. In general, rural households have lower education years and weaker environmental risk awareness than urban residents. Horiguchi [46] noted that farmers are more likely to be exposed to cadmium than other residents in cadmium-contaminated areas. Therefore, rural households in higher heavy metal pollution risk areas are considered as our research objects. The data derived from a survey for rural farmers in heavy metal polluted provinces in China, and the collection took place in November 2018. First, two typical provinces were selected from several main heavy metal polluted provinces based on their emissions. Taking cadmium pollutant as an example, Hunan province and Jiangxi province were chosen. According to China Statistical Yearbooks, the emissions of cadmium pollutant from wastewater in Hunan province and Jiangxi province were 4593.2 and 2092.9 kg, respectively, ranking as the top two provinces in 2015. Besides, news related to cadmium pollutant has been reported in both provinces, arousing widespread concern (see reference [25,47]).

In each selected province, Changde city, Yiyang city, Zhuzhou city of Hunan province, Ganzhou city, and Xinyu city of Jiangxi province were randomly selected. To prevent the existing heavy metal pollution control pilots from interfering with the farmers’ choices, all pilot counties were excluded from the sampling. In total, four villages within each selected county were chosen. About 15 to 20 farmers were then randomly sampled from a list of farming families in each village. The survey covered more than 400 households, and 399 valid conservations were retained after removing the incomplete and unreasonable questionnaires. Since a total of 399 respondents conducted eight choice sets and chose from three options, we obtained 9576 observations.

The specific variables and descriptive statistics are shown in Table 3. The sample has 87% male farmers with an average age of 57, which matches the fact that most agricultural workers are older people in rural China. In terms of scientific knowledge, 19.8% of residents know the ways in which cadmium is ingested, followed by 18.7% who learn the sources of cadmium pollution. However, the health hazards caused by cadmium are poorly understood, and only accounts for 0.5%. The cadmium in their drinking water is perceived to exceed the safety standard by 14.8% of respondents, and 26.6% of the sample has relatives, friends, and neighbors who think they should buy a water purifier with the heavy metal filter to reduce the health risks of cadmium. Also, 29.6% of respondents claim that household water consumption decision is up to themselves. The socio-economic variables include individual characteristics and household characteristics. Studies have shown that gender, age, education, family size, and income play a critical role in consumption decisions [14,48]. The individual characteristics are measured by the gender, age, and education attainment of each respondent. The cultivated land, family size, household per capita income, and the proportion of kids constitute the household characteristics. These characteristics are combined with the defensive attribute and *ASC* variable in robustness checks to investigate the effects of socio-economic factors on the preference for defensive consumption goods.

## 6. Empirical Estimation and Discussion

### 6.1. Discrete Choice Experiment on Water Purifier

#### 6.1.1. Basic Results

In these regressions, we specify the ASC and price as fixed, and the parameters of other attributes as random to follow a normal distribution [49]. The test method proposed by Hausman and McFadden [50] is used to test the IIA hypothesis, and the results show that the IIA hypothesis cannot be supported. Therefore, the mixed logit model is the appropriate method based on the equation (4). The results of preferences for the water purifier with different attributes are reported in Table 4. The models of two samples work well and the Wald tests are significant at the 1% level. In the total sample, the order of rural farmers’ valuation for the three types of water purifier attributes is as follows: warranty period (35.81%), price (14.52%), and free installation (13.63%). Meanwhile, the heavy metal filter will increase 35.81% if a warranty period is provided. The heavy metal filter will increase 14.52% if a price exists. If farmers receive free installation, the heavy metal filter will increase 13.63%.

The standard deviations of these parameter distributions suggest that all attributes on a water purifier have preference heterogeneity except the warranty period. The reason is that the estimated coefficient of warranty period is positive significantly while its standard deviation is not significant. It can be expected that the warranty period of water purifier is necessary for all respondents. The overall results in first two columns show that free installation and heavy metal filter are positive while the coefficient of price is negative at 1% statistical level. It proves that the combination of diversification attributes on defensive consumption goods is preferred by farmers. The survey respondents gain utility if being provided with a water purifier of low price, free installation, longer warranty period, and a heavy metal filter. The standard deviations of these three attributes (price, free installation, and heavy metal filter) are also significant, indicating that not all the rural farmers think they are necessary.

Some farmers in the sample have bought water purifiers before the survey, and we are therefore confined to those families who have not bought a water purifier. These results are reported in the latter two columns of Table 4. For farmers who have not bought water purifiers, the difference in regression results is that the estimated coefficient of brand is positive at 5% significance level. This result proves that farmers who have not bought water purifiers prefer to choose a water purifier with an international brand. Overall, the preferences for a heavy metal filter are positive and significant for both total sample and those who have not bought a water purifier. These coefficients reflect that whether farmers have bought a water purifier or not, there is no difference in their preferences for the defensive attribute.

#### 6.1.2. Extended Analysis on Region Heterogeneity and Substitutability/Complementarity of Attributes

These attributes interacted with a region dummy (whether it is in Hunan province or not) which are included to capture regional heterogeneity of preference in the first two columns of Table 5. Those interaction terms on price, brand, and heavy metal filter are negative at 1% significance level, indicating farmers in Jiangxi province show more preference on them. Ishrat et al. [51] also highlighted the importance of scale heterogeneity in air travel ticket using DCEs. Moreover, the proposed heavy metal filter labels interacted with a province dummy which shows a negative effect, in line with Tang and Zhang [14] that demonstrate the residence location of respondents and affects how the corresponding health risks and attributes are perceived by themselves. The reason these attributes have provincial preference differences is probably due to an information exposure of heavy metal pollution and consumption risks in Jiangxi province just before our interview. In November 2017, several media reported that cadmium and arsenic in farmland soil, irrigation water sources, and rice exceeded national safety standards in Jiujiang city, Jiangxi province.

Considering the complementarity and substitutability between different attributes, especially for the defensive attribute, several interaction terms between them are added in the last two columns of Table 5. The interaction term between a heavy metal filter and a brand is significantly positive, which indicates that there is significant complementarity between the defensive attribute and brand. The attribute of brand is speculated to guarantee the quality and defensive performance of a heavy metal filter to some extent. The coefficients of heavy metal filter and brand as controlled variables are also positive significantly. This result suggests that the heavy metal filter can not only cooperate with brand, but also work alone in improving farmers’ preferences for defensive consumption goods. Besides, the estimated coefficients show that heavy metal filter attribute has a complementarity effect with *Price* attribute, while having a substitution effect on *Installation* attribute. There is no significant complementary or substitutive effect between the heavy metal filter and warranty period. Overall, the defensive attribute shows significant complementarity and substitutability effects on some non-defensive attributes (such as brand, price, and free installation) for a defensive consumption good.

### 6.2. Scientific Knowledge Effects on the Defensive Attribute Preference

The effect of scientific knowledge on preference for the defensive attribute is explored in Table 6. The Wald test in two models are 8800.31 and 9076.04 at 1% significance level for both total samples and those who have not bought a water purifier. The results of three scientific knowledge variables (Source, Intake, and Disease) interacting with a heavy metal filter are not statistically significant. This implies that the scientific knowledge effect of environmental hazards cannot improve farmers’ defensive awareness and preferences in rural areas. Information activities, training programs, and educational interventions can improve understanding and knowledge, but do not necessarily affect behavior [16]. If there are cognition illusions and optimistic bias about the actual environment, people will not prefer defensive labels despite their knowledge of environmental risks [52]. Stoutenborough et al. [1] also noted that scientific knowledge does not result in overwhelming policy support for nuclear power risks. The coefficients of other attributes are consistent with those in Table 4 after scientific knowledge interactions are controlled, thus enhancing the robustness of the above findings.

### 6.3. Risk Perception Effects on the Defensive Attribute Preference

#### 6.3.1. Regression Results

Table 7 reports the effects of risk perception on farmers’ preferences for a water purifier with the defensive attribute. The two models run well as a whole, and the Wald test results are also significant. The estimated coefficients of farmers’ agreement on “cadmium in your drinking water exceeds the safety standard” (attitude) are not statistically significant in two samples. This means the risk attitude perception on drinking water may not increase their preferences for the defensive attribute. A positive sign of preference to the neighborhood norm perception measured by “farmers have relatives, friends, and neighbors who think they should buy a water purifier with the heavy metal filter to reduce the health risks of cadmium” (norm) implies that farmers are more likely to choose defensive consumption goods affected by their neighborhood norm perception. This result is in general congruence with the conclusions of Ortega et al. [22] regarding the risk perception effects on consumers’ willingness to pay in response to food safety information. Some literature also found that consumers’ social norm perception has a positive effect on their environmental attitudes and behaviors [37,42]. Regarding the model that interacted with behavioral control perception, we find nonsignificant coefficients between “the drinking water source in the household is decided by themselves (control)” and farmers’ preferences for the defensive attribute. In conclusion, the risk perception of neighborhood norm is a significant factor in farmers’ preference for defensive consumption goods. This hypothesis is confirmed in the literature by Heiman and Lowengart [36] and Janssen and Hamm [53], according to whom, the decision-making process depends more on the risk perception rather than objective knowledge, especially for negative information about health risks. As the news of heavy metal pollution risk reported in Jiangxi province may enhance farmers’ risk awareness (We have referred it in Section 6.1.2.), this finding also echoes the result in Table 5 that farmers in Jiangxi province prefer the defensive attribute more compared with Hunan province. The feelings of environment and situations play an important role in their risk perception and decision-making [54].

#### 6.3.2. Interaction with Income Gap and Education Gap

The paper aims to analyze the heterogeneous effects of neighborhood norm perception on a farmer’s preference for the defensive attribute. Farmers with large income gaps or education gaps are more likely to be influenced by others when making decisions. Several interaction terms with average income gaps and average education gaps are added to the regressions to capture the possible heterogeneity effects in Table 8 and Table 9. Table 8 reports that the interacted coefficients between the neighborhood norm perception, heavy metal filter, and average income gap (Incgap) are significantly positive. It proves that farmers can enhance preferences for the defensive attribute affected by neighborhood norm perception as the income gap narrows. Existing studies have pointed out that low income residents usually turn to their immediate social network of friends and family for health advice or risk prevention experience, rather than health experts or official information sources [45,55]. Our finding is echoed by Williams, [5] that wealthier people usually take the least time-consuming avoidance behaviors, such as buying water treatment devices. These coefficients are robust to both the average income gap and the absolute value of the average income gap.

As for the average education gap in Table 9, the interacted coefficients with the neighborhood norm perception are not statistically significant. It reflects that average education gap is not an effective factor for illustrating the neighborhood norm perception effect on the farmers’ preferences for the defensive attribute. Some literature has found that education attainment is beneficial to improve health awareness and defensive behaviors significantly [6,56,57]. However, Visschers et al. [56] suggested that nutrition education cannot stimulate the information use behaviors by all types of consumers. This result proves that both average education gap and the absolute value of average education gap do not appear to be effective in interacting the neighborhood norm perception effect on preference for the defensive attribute on defensive consumption goods.

### 6.4. Robustness Checks

#### 6.4.1. Robustness Checks for Basic Results

The first two columns of Table 10 report the basic estimation results after the interaction terms between some family characteristics (the proportion of the elderly and the proportion of children) and attributes are included. The order of marginal substitution rates for warranty period, price, and free installation are the same as those in Table 4, which are confirmed to be robust. The elderly and children are special groups that are vulnerable to environmental risks in the family. The interacted coefficients of elders and children with RO are both not significant. It indicates that family characteristics (such as elderly and children) have no heterogeneous preference effect for the defensive attribute on farmers, while Johnstone and Serret [4] have found that the decision to buy a water purification is affected by children in the family. The interaction terms between *ASC* and all the individual characteristics (including gender, age, education attainment, self-evaluation health, family size, and per capita income) are shown in column (3). These coefficients are also not statistically significant, which further confirms that individual characteristics do not impose a significant heterogeneous preference effect on defensive consumption goods. The conditional logit model (CLM) result that replaced mixed logit model is presented in the last column. These coefficients are provided as comparison since the Hausman and McFadden test for the IIA property imposed by CLM cannot hold effectively. The fit goodness of this model is usually judged by Pseudo R^2^. The model has better fitting effect when the Pseudo R^2^ is between 0.2 and 0.4. The value of Pseudo R^2^ for CLM is between the recommended values in this paper. It indicates that farmers are found to prefer water purifiers with low price, free installation, longer warranty period, and heavy metal filter. The results are consistent with the mixed logit model in Table 4, which enhances the robustness of our findings.

#### 6.4.2. Robustness Checks for Scientific Knowledge and Risk Perception Effect Results

Table 11 and Table 12 report the results of preference for the defensive attribute after the interaction term of a scientific knowledge variable or risk perception variable is controlled separately in each regression. It should be noted that the preference coefficient interacted with neighborhood norm perception is still positive at 1% significance level, while not significant for other interaction terms. The significance and direction are basically consistent with the results in Table 6 and Table 7, which enhances the stability of our results. Besides, as for interaction regressions with average income gap or average education gap, our findings are also robust to those that interact with the upper/lower quartile of income gap and education gap (not shown).

Some studies have noted that scientific knowledge may affect public risk evaluations [1,44]. The paper also examines whether there is a significant interacted effect between scientific knowledge, risk perception, and preference for the defensive attribute and the estimated coefficients are not significant (not shown).

## 7. Conclusions

Based on a microeconomic survey data of rural farmers in Hunan and Jiangxi provinces in China, a DCE method was employed to investigate their preferences for the water purifier as a defensive consumption good. Using the mixed logit model, farmers’ preference for the defensive attribute subject to scientific knowledge and risk perception effects were investigated, respectively, under the background of heavy metal pollution risk. We also discussed whether the average income gap and average education gap interact with the relationship between scientific knowledge/risk perception and preference for the defensive attribute. The main conclusions drawn in this paper are listed as follows.

First, lower price, free installation, longer warranty period, and heavy metal filter are significant factors of preference for water purifiers under heavy metal pollution risk. Region heterogeneity is observed in farmers’ preferences for the defensive attribute, while family characteristic heterogeneity is not found. The defensive attribute is complementary to the brand and price, but substitutable to free installation significantly. Second, an individual’s risk perception of neighborhood norm dominates the preference for defensive consumption goods while the scientific knowledge effect of heavy metal pollution risk is nonsignificant. Specifically, in risk perception, farmers’ preferences for the defensive attribute are not affected by risk attitude perception and behavioral control perception effects. Third, the neighborhood norm perception effect on farmers’ preferences for the defensive attribute are significantly affected by their average income gaps. In other words, farmers’ neighborhood norm perception can enhance their preferences for the defensive attribute as average income gaps narrow.

In the future, efforts can be made to promote defensive consumption behaviors to resist heavy metal pollution and health risks, since complete environmental recover and repair will not be fulfilled in a short time. Several policy implications are drawn and combined with the above findings. First, an improvement in the training on self-defense against environmental risks are needed to exert a greater impact on the defensive awareness of potential health risks, especially for residents living in high environmental risk areas. Second, the need for disseminating health and environmental defensive information through informal social networks should be emphasized more in defensive policy constructions, especially for low-income residents. Third, the water purifier as a complementary drinking water appliance implies an additional financial burden and defensive expenditures [23]. Options such as preferential services support for defensive consumption goods maintenance can be considered. The government should further strengthen the quality monitoring of water plants to meet the water safety standards, especially in rural areas. It should be the focus of the government’s work on the treatment of heavy metal pollution and green development of cleaner production.

## Figures and Tables

**Table 1:** Attributes and levels of a water purifier.

Attributes	Definition	Level
Price	Market price (Continuous)	1000 CNY 2000 CNY 3000 CNY
Brand	Whether it is an international brand (Dummy)	Yes, No
Free installation	Whether your home is located within the free installation covered areas (Dummy)	Yes, No
Warranty period	Warranty period (Continuous)	1 Year 2 Years 3 Years
Heavy metal filter	Whether it has RO reverse osmosis membrane that can remove heavy metal pollutants (Dummy)	Yes, No

**Table 2:** Example of a choice set.

NO.	Asking Order	Option	Price	Brand	Installation	Repair	RO
a	2	A	1000	No	Yes	2 Years	Yes
		B	2000	Yes	No	1 Years	No
		C	Neither				

**Table 3:** The definition of variables and summary statistics.

Variables	Variable Definition and Value	Mean	Standard Deviation
Scientific Knowledge Variables (Binary variables of whether correctly answered)			
Source	Where do you think cadmium pollution in the soil is mainly from (A. Industrial and mining B. agricultural production C. atmospheric subsidence)	0.187	0.463
Intake	How do you think the body ingests cadmium (A. food B. drinking water C. air)	0.198	0.513
Disease	What outpatient department should you visit if you get sick from cadmium ingestion *	0.005	0.070
Risk Perception Variables			
Attitude	The cadmium in your drinking water exceeds the safety standard (Yes = 1, No = 0)	0.148	0.355
Norm	You have relatives, friends and neighbors who think you should buy a water purifier with the heavy metal filter to reduce the health risks of cadmium (Yes = 1, No = 0)	0.266	0.442
Control	The household water consumption decision is up to you (Yes = 1, No = 0)	0.296	0.457
Household Head Characteristic Variables			
Gender	Male = 1, Female = 0	0.872	0.581
Age	Age of each respondent (years)	57.987	10.209
Health	Self-identified health status (0 = loss of labor ability, 1 = poor, 2 = medium, 3 = good, 4 = excellent)	2.805	0.992
Size	Family size	4.679	2.185
Income	Household per capita annual income (1000 CNY)	14.011	24.329
Kid	The number of kids (<18 years old) / family size	0.272	0.313
Land	Total cultivated land (mu)	54.129	279.98
Interacted Variables			
Incgap	The absolute value of difference between an individual’s household per capita income and average household per capita income at village level (10^3^ CNY)	1.214	0.070
Edugap	The absolute value of difference between an individual’s education years and average education years at village level (years)	2.383	1.889

* Options: pediatrics, gynecology (production), ophthalmology, dermatology, otolaryngology, dental, regions, acupuncture and massage, respiratory medicine and digestive diseases, urinary medicine, cardiology, onset, endocrinology and neurology, pediatrics, infection, general surgery, orthopedics, neurosurgery, liver and gallbladder surgery, kidneys and urinary surgery, acute, and oncology, acupuncture and massage, psychological consulting room.

**Table 4:** The results of mixed logit model.

Variables	Total Sample		Have not Bought Sample	
	Mean	S.D.	Mean	S.D.
ASC	26.430 ***	-	26.340 ***	-
Price	−1.969 ***	-	−1.718 ***	-
Brand	0.025	0.886 ***	0.166 **	0.773 ***
Installation	0.185 ***	0.425 ***	0.146 **	0.492 ***
Repair	0.486 ***	0.019	0.496 ***	−0.019
RO	1.357 ***	1.148 ***	1.348 ***	1.150 ***
Wald Chi2	9156.58 ***		8993.32 ***	
Observations	9576		6960	

Note: ** significant at 5% level; *** significant at 1% level.

**Table 5:** The substitutability and complementarity of attributes and region heterogeneity results.

Variables	Region Heterogeneity		Substitutability and Complementarity	
	Mean	S.D.	Mean	S.D.
ASC	26.175 ***	-	28.887 ***	-
Price	−0.275	-	−12.024 ***	-
Brand	−0.100	0.891 ***	2.392 ***	0.674 ***
Installation	0.184 *	0.446 ***	2.405 ***	0.288 **
Repair	0.618 ***	0.010	1.720 ***	−0.073
RO	1.777 ***	1.129 ***	0.785 **	1.214 ***
Price × Province	−0.658 ***	-	-	-
Brand × Province	−0.255 ***	-	-	-
Installation × Province	0.187	-	-	-
Repair × Province	0.008	-	-	-
RO × Province	−0.199 ***	-	-	-
Price × RO	-	-	−0.398 ***	-
Brand × RO	-	-	0.465 **	-
Installation × RO	-	-	−0.370 **	-
Repair × RO	-	-	−0.178	-
Price × Brand	-	-	0.478 ***	-
Price × Installation	-	-	0.563 ***	-
Price × Repair	-	-	0.486 ***	-
Brand × Installation	-	-	−0.368	-
Brand × Repair	-	-	−0.831 ***	-
Installation × Repair	-	-	−0.461 ***	-
Wald Chi2	7868.23 ***		9494.33 ***	
Observations	9576		9576	

Note: * Significant at 10% level; ** significant at 5% level; *** significant at 1% level.

**Table 6:** The results of scientific knowledge effects on the defensive attribute preference.

Variables	Total Sample		Have not Bought Sample	
	Mean	S.D.	Mean	S.D.
ASC	25.907 ***	-	26.353 ***	-
Price	−1.962 ***	-	−1.713 ***	-
Brand	0.027	0.884 ***	0.169 **	0.771 ***
Installation	0.187 ***	0.424 ***	0.146 **	0.493 ***
Repair	0.486 ***	0.019	0.495 ***	−0.020
RO	1.410 ***	1.145 ***	1.377 ***	1.147 ***
RO × Source	−0.235	-	−0.194	-
RO × Intake	0.137	-	0.150	-
RO × Disease	−0.136	-	−0.256	-
Wald Chi2	8800.16 ***		9076.04 ***	
Observations	9576		6960	

Note: ** significant at 5% level; *** significant at 1% level.

**Table 7:** The results of risk perception effects on the defensive attribute preference.

Variables	Total Sample		Have not Bought Sample	
	Mean	S.D.	Mean	S.D.
ASC	28.547 ***	-	25.692 ***	-
Price	−1.974 ***	-	−1.729 ***	-
Brand	0.004	−0.896 ***	0.165 **	0.774 ***
Installation	0.187 ***	0.426 ***	0.146 **	0.483 ***
Repair	0.489 ***	−0.006	0.497 ***	−0.012
RO	0.828 ***	1.125 ***	0.794 **	1.125 ***
RO ×Attitude	−0.205	-	−0.277	-
RO × Norm	0.540 ***	-	0.485 **	-
RO × Control	0.202	-	0.306	-
Wald Chi2	10699.60 ***		8289.45 ***	
Observations	9576		6960	

Note: ** significant at 5% level; *** significant at 1% level.

**Table 8:** The results of mechanisms on the average income gap.

Variables	Absolute Value of Income Gap		Average Income Gap	
	Mean	S.D.	Mean	S.D.
ASC	27.115 ***	-	26.641 ***	-
Price	−1.995 ***	-	−1.983 ***	-
Brand	0.023	0.899 ***	0.024	0.895 ***
Installation	0.187 ***	0.428 ***	0.185 ***	0.420 ***
Repair	0.487 ***	0.015	0.487 ***	0.017
RO	0.702 ***	1.116 ***	1.013 ***	1.116 ***
RO × Incgap × Norm	−0.028 ***	-	−0.026 ***	-
RO × Incgap	0.019 **	-	0.023 ***	-
RO × Norm	0.870 ***	-	0.444 **	-
Wald Chi2	9192.39 ***		9089.73 ***	
Observations	9576		9576	

Note: ** significant at 5% level; *** significant at 1% level.

**Table 9:** The results of mechanisms on the average education gap.

Variables	Absolute Value of Edu Gap		Average Education Gap	
	Mean	S.D.	Mean	S.D.
ASC	26.046 ***	-	26.360 ***	-
Price	−1.993 ***	-	−1.995 ***	-
Brand	0.022	0.894 ***	0.019	0.898 ***
Installation	0.187 ***	0.426 ***	0.187 ***	0.422 ***
Repair	0.487 ***	0.018	0.487 ***	0.018
RO	1.050 ***	1.132 ***	0.922 ***	1.128 ***
RO × Edugap × Norm	0.030	-	−0.031	-
RO × Edugap	−0.058	-	0.077 *	-
RO × Norm	0.477 *	-	0.536 ***	-
Wald Chi2	8773.71 ***		9199.48 ***	
Observations	9576		9576	

Note: * Significant at 10% level; *** significant at 1% level.

**Table 10:** The results of basic model on family and individual characteristics.

Variables	The Elder Interaction Model		Children Interaction Model		ASC and Characteristics Interaction Model		Conditional Logit Model
	Mean	S.D.	Mean	S.D.	Mean	S.D.	Mean
ASC	25.922 ***	-	26.002 ***	-	25.890 ***	-	19.499 ***
Price	−2.146 ***	-	−2.863 ***	-	−1.969 ***	-	−2.203 ***
Brand	0.034	0.887 ***	0.010	0.905 ***	0.025	0.886 ***	0.030
Installation	0.183 **	0.427 ***	0.212 **	0.430 ***	0.185 ***	0.425 ***	0.233 ***
Repair	0.470 ***	0.018	0.441 ***	0.020	0.486 ***	0.019	0.513 ***
RO	1.281 ***	1.145 ***	1.438 ***	1.158 ***	1.357 ***	1.148 ***	1.525 ***
Price × Elder	0.096	-	-	-	-	-	-
Brand × Elder	−0.046	-	-	-	-	-	-
Installation × Elder	0.021	-	-	-	-	-	-
Repair × Elder	0.086	-	-	-	-	-	-
RO × Elder	0.402	-	-	-	-	-	-
Price × Children	-	-	0.301 ***	-	-	-	-
Brand × Children	-	-	0.060	-	-	-	-
Installation × Children	-	-	−0.107	-	-	-	-
Repair × Children	-	-	0.162	-	-	-	-
RO × Children	-	-	−0.261	-	-	-	-
ASC × Gender	-	-	-	-	0.019	-	-
ASC × Age	-	-	-	-	−0.039	-	-
ASC × Edu	-	-	-	-	0.085	-	-
ASC × Health	-	-	-	-	0.059	-	-
ASC × Land	-	-	-	-	−0.017	-	-
ASC × Size	-	-	-	-	0.067	-	-
ASC × Income	-	-	-	-	−0.304	-	-
Wald Chi2	8993.66 ***		8593.12 ***		10220.18 ***		52624.52 ***
Pseudo R2	-		-		-		0.243
Observations	9576		9576		9576		9576

Note: ** significant at 5% level; *** significant at 1% level.

**Table 11:** The robustness check results of scientific knowledge effects.

Variables	Source Interaction		Intake Interaction		Disease Interaction	
	Mean	S.D.	Mean	S.D.	Mean	S.D.
ASC	25.911 ***	-	26.436 ***	-	26.432 ***	-
Price	−1.965 ***	-	−1.969 ***	-	−1.968 ***	-
Brand	0.026	0.885 ***	0.025	0.886 ***	0.025	0.885 ***
Installation	0.186 ***	0.422 ***	0.185 ***	0.425 ***	0.186 ***	0.424 ***
Repair	0.486 ***	0.018	0.486 ***	0.018	0.486 ***	0.018
RO	1.427 ***	1.145 ***	1.369 ***	1.148 ***	1.360 ***	1.148 ***
RO × Source	−0.122	-	-	-	-	-
RO × Intake	-	-	−0.018	-	-	-
RO × Disease	-	-	-	-	−0.202	-
Wald Chi2	8663.90 ***		9129.81 ***		9161.38 ***	
Observations	9576		9576		9576	

Note: *** significant at 1% level.

**Table 12:** The robustness check results of risk perception effects.

Variables	Attitude Interaction		Norm Interaction		Control Interaction	
	Mean	S.D.	Mean	S.D.	Mean	S.D.
ASC	25.796 ***	-	26.437 ***	-	26.458 ***	-
Price	−1.967 ***	-	−1.994 ***	-	−1.970 ***	-
Brand	0.026	0.887 ***	0.023	0.894 ***	0.024	0.885 ***
Installation	0.187 ***	0.430 ***	0.186 ***	0.426 ***	0.185 ***	0.425 ***
Repair	0.486 ***	0.020	0.487 ***	0.017	0.486 ***	0.019
RO	1.443 ***	1.144 ***	0.922 ***	1.130 ***	1.132 ***	1.146 ***
RO × Attitude	−0.192	-	-	-	-	-
RO × Norm	-	-	0.537 ***	-	-	-
RO × Control	-	-	-	-	0.251	-
Wald Chi2	8544.76 ***		8899.71 ***		9164.56 ***	
Observations	9576		9576		9576	

Note: *** significant at 1% level.
